# The ABC Transporter Components HgdB and HgdC are Important for Glycolipid Layer Composition and Function of Heterocysts in *Anabaena* sp. PCC 7120

**DOI:** 10.3390/life8030026

**Published:** 2018-07-02

**Authors:** Dmitry Shvarev, Carolina N. Nishi, Lars Wörmer, Iris Maldener

**Affiliations:** 1 Organismic Interactions, Interfaculty Institute of Microbiology and Infection Medicine, Eberhard Karls University of Tübingen, 72076 Tübingen, Germany; dmitryshvarev@gmail.com (D.S.); nishi.carolina@gmail.com (C.N.N.); 2 Organic Geochemistry Group, MARUM—Center for Marine Environmental Sciences, University of Bremen, 28359 Bremen, Germany; lwoermer@marum.de

**Keywords:** cyanobacteria, *Anabaena*, heterocyst, glycolipid, ABC transporter, *hgdB*, nitrogen fixation

## Abstract

*Anabaena* sp. PCC 7120 is a filamentous cyanobacterium able to fix atmospheric nitrogen in semi-regularly spaced heterocysts. For correct heterocyst function, a special cell envelope consisting of a glycolipid layer and a polysaccharide layer is essential. We investigated the role of the genes *hgdB* and *hgdC*, encoding domains of a putative ABC transporter, in heterocyst maturation. We investigated the subcellular localization of the fusion protein HgdC-GFP and followed the differential expression of the *hgdB* and *hgdC* genes during heterocyst maturation. Using a single recombination approach, we created a mutant in *hgdB* gene and studied its phenotype by microscopy and analytical chromatography. Although heterocysts are formed in the mutant, the structure of the glycolipid layer is aberrant and also contains an atypical ratio of the two major glycolipids. As shown by a pull-down assay, HgdB interacts with the outer membrane protein TolC, which indicates a function as a type 1 secretion system. We show that the *hgdB-hgdC* genes are essential for the creation of micro-oxic conditions by influencing the correct composition of the glycolipid layer for heterocyst function. Our observations confirm the significance of the *hgdB-hgdC* gene cluster and shed light on a novel mode of regulation of heterocyst envelope formation.

## 1. Introduction

*Anabaena* sp. PCC 7120 (also known as *Nostoc* sp. PCC 7120; hereafter, *Anabaena*) is a filamentous fresh water cyanobacterium. When growing in the presence of combined nitrogen compounds, its trichomes consist of vegetative photosynthetic cells. Upon depletion of a nitrogen source *Anabaena* begins a developmental program leading to formation of nitrogen-fixing cells called heterocysts, which appear in a semi-regular pattern within the trichome [1,2,3,4]. The heterocysts contain the highly oxygen-sensitive nitrogenase complex, which performs nitrogen fixation. In order to protect the nitrogenase from oxygen, heterocysts carry out different strategies. At the metabolic level, they inactivate oxygenic photosynthesis and increase the respiration rate to remove oxygen and to meet the demand of the nitrogenase for energy. Morphological changes precede these metabolic alterations, especially the early formation of an additional cell envelope, which protects these cells from environmental oxygen. This special envelope consists of two layers: the outer exo-polysaccharide layer (hep layer) and the inner laminated glycolipid layer (hgl layer). The hgl layer is the actual barrier for gases, including oxygen, while the outer hep layer mechanically protects the subjacent hgl layer [2]. The hgl layer is composed of so-called heterocyst-specific glycolipids (HGLs), and there are several HGLs present in the heterocyst forming cyanobacteria. They differ in the length of the carbon chain, the type and number of functional groups (e.g., diol, keto-ol, triol, keto-diols), and the sugar moiety [5,6,7,8,9,10,11,12]. The two major HGLs in *Anabaena* are characterized as 1-α-glucosyl-3,25-hexacosanediol and its 3-ketotautomer [8]. In recent years, several open reading frames (orfs) have been identified, which are related to hgl layer formation, including genes encoding enzymes for the synthesis of HGLs as well as for their deposition (summarized in [2]).

After the hep layer is formed, the glycolipids are transferred through the gram-negative cell wall by transport systems, structurally similar to type 1 secretion systems (T1SS). These are widely distributed among gram-negative bacteria. Usually they comprise the oligomeric outer membrane protein (OMP) TolC and an oligomeric periplasmic membrane fusion protein (MFP), which connects an OMP with an inner membrane factor (IMF), another component of the T1SS. Typically, OMPs with MFPs form a pore that allows different substrates to be transferred through the gram-negative cell wall, while an IMF provides the energy for the whole complex to work [13,14]. IMFs can be divided into three major protein superfamilies, which differ in their manner of gaining energy; these are the ATP-dependent ABC superfamily (ATP-binding cassette) and two H+-driven superfamilies, RND (resistance–nodulation–division) and MFS (major facilitator) [14]. In fact, ABC transporters are widely distributed in *Anabaena*, where they fulfill different functions, including export and import of various chemical compounds and nutrients, to name a few. Indeed, *Anabaena* ABC transporters in general also participate in heterocyst development and nitrogen fixation [15].

A functional T1SS-like efflux pump in *Anabaena*, containing an ABC transporter and performing export of HGLs into the heterocyst cell envelope, has been identified previously [16,17]. This efflux pump consists of the TolC homologue OMP HgdD [18,19], the MFP DevB, and the DevCA ABC transporter [20,21]. The MFP DevB shows a sequence similarity to other MFPs from different organisms (for instance, it shares 28% identity and 67% coverage with MacA from *Escherichia coli* according to the NCBI BLAST online tool). The ABC transporter DevCA comprises an IMF (permease DevC) and a separate ATPase (DevA). These two proteins, when artificially fused together, show a significant sequence similarity to the protein MacB (35% identity, 68% coverage according to BLAST), a monomer of a dimeric macrolide exporter component consisting of a permease and an ATPase fused together. HgdD presumably represents a trimer, DevB is probably a hexamer, and DevC and DevA form dimers. The interactions between the subunits and their stoichiometry were confirmed *in vitro*, and the activity of the pump *in vitro* was at the highest level when using HGLs as a substrate [16].

Although it has been shown that the efflux pump HgdD-DevBCA serves as a transporter of HGLs, it is still unknown how HGLs are arranged in the laminated layer, which proteins assist the export and arrangement of HGLs, and whether any other transporters are involved in any of these processes. Previous work demonstrated a large variety of proteins involved in heterocyst development in *Anabaena*, particularly in heterocyst envelope formation [22,23]. Also, several putative transporters, which are homologous to the DevBCA transporter, may play a role in this process. For instance, proteins All0807, All0808, and All0809 with HgdD also may constitute a pump with a still unknown substrate, but this transporter is also necessary for heterocyst function and diazotrophic growth of *Anabaena* [24]. Bioinformatic search revealed several additional homologues of DevBCA with unknown functions, and these proteins may play a role in the correct development of the heterocyst cell envelope: All5347/6, Alr3647/8/9, All2651/2, Alr4280/1/2, and Alr4973/4/5 [25].

Previously, Fan and colleagues [22] described a cluster of genes (*all5343–all5359*) involved in synthesis and deposition of heterocyst glycolipids. Mutants in orfs *all5347*, *all5346*, and *all5345* were affected in deposition of the HGLs and hence the genes were denoted as *hgdB*, *hgdC*, and *hgdA*, respectively (hgd for heterocyst glycolipid deposition).

**Figure 1:**
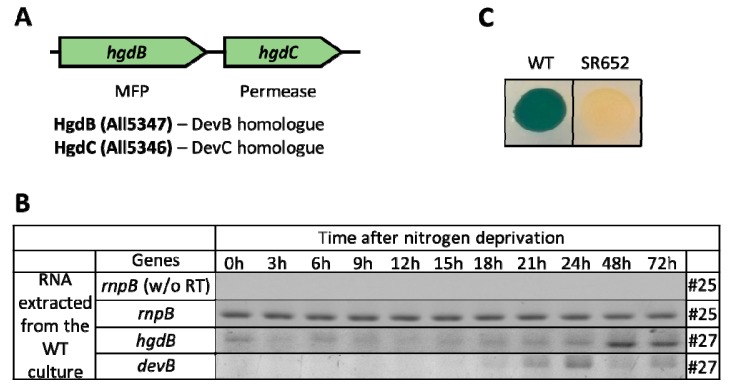
Expression of the *hgdB* gene and growth of the mutant strain SR652. (**A**) Scheme showing the *hgdBC* (*all5347/6*) gene cluster. MFP—membrane fusion protein; (**B**) Time-dependent expression analysis of *hgdB* in comparison to *devB* in the wild-type (WT) culture during nitrogen starvation performed by RT-PCR. *rnpB* (ribonuclease B) was used as a loading control ensuring the equal amount of RNA used for cDNA synthesis in each sample. Numbers at the right side of the table are numbers of PCR cycles. (**C**) growth of spotted colonies from a nitrogen stepdown experiment of liquid cultures of the WT and SR652 strain on BG11_0_ agar plates, starting at 0.25 μg of chlorophyll *a* and incubated for 10 days.

In this work, we focused on the functions of the *devBC* homologues [22] *hgdB* (*all5347*) and *hgdC* (*all53*46) (Figure 1A). HgdB and HgdC proteins show a high sequence similarity to DevB and DevC, respectively, which is well illustrated by their alignment using Multalign software [26] (Figure S1). The *hgdA* gene encodes a putative oxidoreductase/epimerase, and therefore is not a component of a DevBCA-like export system. Interestingly, the gene cluster is devoid of a gene encoding an ATPase, which is needed for normal functioning of an ABC transporter. Here, we performed mutational analysis of the *hgdB* gene to examine its role in hgl layer formation in more detail. We confirmed the aberrant mutant cell envelope, resulting in the loss of nitrogen fixation under aerobic growth conditions. In addition, we present the cellular localization of the permease encoded by the *hgdC* gene and discuss the putative function of the HgdBC complex during heterocyst development in *Anabaena*.

## 2. Materials and Methods

**Organisms and growth conditions.***Anabaena* wild type (WT) and its derivative mutant strains were cultivated in liquid BG11 medium [27] in 100-mL Erlenmeyer flasks under continuous illumination (17–22 μmol photons m^−2^ s^−1^) at 28 °C with shaking at 120 rpm. With the purpose of RNA isolation for gene expression studies, one-liter bubbling bottles with 700 mL of nitrate-free BG11 medium, supplemented with 5 mM NH_4_Cl as the nitrogen source and 5 mM of TES buffer (pH7.8), were used. During cultivation, bottles were continuously supplied with CO_2_-enriched air (2%). Spectinomycin and streptomycin (2.5 μg mL^−1^ each) were added to BG11 media for growing the mutant strains.

For the nitrogen stepdown experiments, the strains were washed three times in BG11 medium lacking NO_3_^−^ and cultivated in the same medium.

All of the cloning and plasmid maintenance was performed using *E. coli* strains Top10, NEB10, Lemo21 (DE3), and HB101. For the triparental mating, *E. coli* strain J53 (bearing the conjugative plasmid RP4), strain HB101 (bearing the helper plasmid pRL528 and the cargo plasmid), and the *Anabaena* wild type were used [28,29] (Tables S1–S3).

Overexpression of proteins for the pull-down assay was performed in *E. coli* Lemo21 (DE3) strain (Table S2).

**DNA manipulation.** To create an insertional mutant of *hgdB* by single recombination, an internal fragment of the gene was amplified by colony PCR from the wild-type *Anabaena* culture (see Table S4 for primers) and cloned into the *Xho*I restricted suicide vector pRL277 (Table S3) using Gibson assembly [30], (see also Figure S2A). The resulting plasmid pIM652 was transferred into wild-type *Anabaena* cells using triparental mating, followed by selection on streptomycin- and spectinomycin-containing BG11 agar plates. In the resistant *Anabaena* colonies, the *hgdB* gene was disrupted by the pRL277 vector. Complete segregation of one selected mutant colony was confirmed by colony PCR (Figure S2B), and the strain was named SR652.

**Figure 2:**
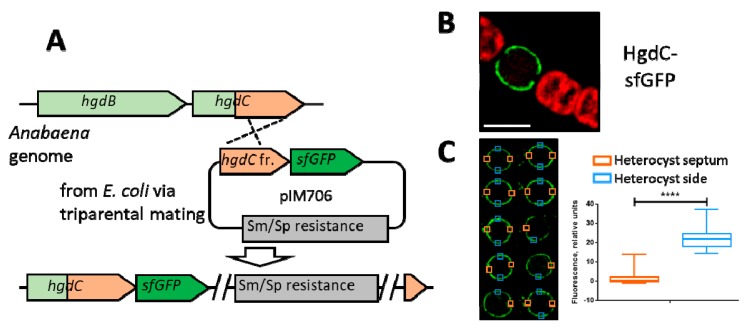
Subcellular localization of HgdC-GFP. (**A**) Scheme showing the homologous recombination approach used to obtain the C-terminal HgdC super-folder GFP (sfGFP) fusion; *hgdC* fr.—fragment of *hgdC* corresponding to the protein’s C-terminus. (**B**) Fluorescent micrographs of *Anabaena* filaments bearing translational fusions of the gene *hgdC* (*all5346*) with *sfGFP* after three days of nitrogen starvation. The green color corresponds to the fluorescence of GFP; the red color indicates the cyanobacterial autofluorescence. Bar, 5 μm. (**C**) Quantification of GFP at defined positions in the heterocyst wall. The left panel shows the analyzed heterocysts. Blue and orange boxes indicate areas used for quantification by GelQuant.NET software provided by biochemlabsolutions.com (represented in the graph on the right). Fluorescence intensity (RU—relative units) was analyzed for 10 heterocysts from different filaments, as depicted in the left panel. Student’s *t* test *P* value is *P* < 0.0001 (****).

For localization studies, a plasmid with a gene coding for a translational fusion of the HgdC C-terminal end with the super-folder GFP (sfGFP) [31] was constructed. Sequences of the 3′ end of *hgdC* and the entire *sfGFP* were amplified by PCR and cloned into the *Xho*I restricted suicide vector pRL277 using Gibson assembly. This resulting plasmid pIM706 (see Figure 2A) was transferred into wild-type *Anabaena* cells using triparental mating, followed by plating on streptomycin- and spectinomycin-containing BG11 agar plates. Growing *Anabaena* colonies contained the *hgdC* gene fused with the *sfGFP*. The fusion was confirmed by colony PCR, and the strain was called SR706.

**RNA isolation and RT-PCR.** RNA was isolated at different time points after nitrogen stepdown (0 h, 3 h, 6 h, 9 h, 12 h, 15 h, 18 h, 21 h, 24 h, 48 h, 72 h) from wild-type *Anabaena* cells, grown in bubbling bottles (see Section 2 on organisms and growth conditions for details) using UPzol reagent (Biotechrabbit, Henningsdorf, Germany) according to the manufacturer’s instructions. Purity and concentration of the extracted RNA were estimated using RNA electrophoresis and GelQuantNET software provided by biochemlabsolutions.com. The reverse transcription (RT) reactions were performed using the Applied Biosystems RT-reaction kit. Primers for PCR reactions performed with the cDNA (synthesized from the RNA) are listed in the Table S4.

**Staining methods for light microscopy.** BODIPY (borondipyrromethene difluoride 493/503, Molecular Probes, Life Technologies, ThermoFisher Scientific) staining was done using the protocol described by Perez and colleagues [32]. Briefly, 1 mL of *Anabaena* cell suspension was centrifuged at 4000× *g* for 10 min, washed with the PBS buffer and resuspended in 200 μL PBS. 1 μL of 50 ng mL^−1^ BODIPY in DMSO was added. The cell suspension was incubated in the dark for 30 min at room temperature and examined by light and fluorescence microscopy.

Vancomycin-FL (Van-FL; Bodipy FL Conjugate; Invitrogen), the fluorescent derivate of vancomycin, was added to *Anabaena* filaments, and the suspension was incubated in the dark for 30 min before microscopy [33].

To capture fluorescence or phase-contrast images of samples stained with BODIPY or Vancomycin-FL, a Leica (Wetzlar, Germany) DM 5500 B microscope connected to a Leica (Wetzlar, Germany) monochrome DFC360 FX camera was used.

Alcian blue staining was performed using a standard protocol [34]. Alcian blue was dissolved in water, resulting in a 1.5% solution. The solution was mixed with the cell suspension (in a ratio of 1:20) and incubated at room temperature for 5 min.

For the triphenyl tetrazolium chloride (TTC) staining, the cell suspension was mixed with the TTC solution (0.01% of TTC (*w*/*v*) in the final mixture) and incubated in the dark for 10 min at room temperature [35].

Alcian blue- and TTC-stained filaments were examined using a Leica (Wetzlar, Germany) DM 2500 microscope connected to a Leica (Wetzlar, Germany) DFC420C camera.

**Microscopy.** For light and fluorescence microscopy, wild-type and mutant *Anabaena* cells were placed onto agar-covered glass slides. A Leica DM 2500 microscope connected to a Leica DFC420C camera or a Leica DM5500 B microscope connected to a Leica monochrome DFC360 FX camera were used.

Fluorescence of sfGFP, BODIPY, and vancomycin-FL was captured using a BP470 40-nm excitation filter and a BP525 50-nm emission filter. Cyanobacterial autofluorescence was monitored using a 50-nm BP535 excitation filter and a 75-nm BP610 emission filter. Images were exposed for 80 to 150 ms in the fluorescence channels. Images of sfGFP and BODIPY fluorescence were taken as Z-stacks with 0.4-μm intervals. Z-stacks were afterwards used to perform 3D deconvolution using the integrated function of the Leica ASF software (Leica Microsystems). Images of fluorescence were recolored by the Leica ASF software based on the filters used.

For electron microscopy, fixation and post-fixation of cells were performed with glutaraldehyde and potassium permanganate [20]. Ultrathin sections were stained with uranyl acetate and lead citrate. The sections were examined with a Philips (Eindhoven, the Netherlands) Tecnai 10 electron microscope at 80 kHz.

**Analysis of the heterocyst-specific glycolipids.** Thin-layer chromatography was performed as described in [36], with minor modifications. Briefly, cells were pelleted at equal chlorophyll *a*_concentration (measured according to [37]) for the WT and the mutant and resuspended in a methanol:chloroform mixture (1:1). Afterwards the solvents were evaporated in the air under a fume hood. Lipids were dissolved in chloroform and applied on an aluminum plate coated with silica gel (Macherey-Nagel, #818033). Subsequently, thin layer chromatography (TLC) was run in a mobile phase composed of chloroform:methanol:acetic acid:water in a ratio of 23:4:2.7:1. Lipids were visualized by spraying the plate with 25–50% sulfuric acid and exposing it to 180 °C for 60–120 s.

**HPLC-MS of lipid extracts.** For a more precise identification and quantification of the different HGL species, they were analyzed by high performance liquid chromatography coupled to mass spectrometry (HPLC-MS) following Wörmer et al. [38]. Total lipid extracts, obtained as described above for TLC, were investigated after being re-dissolved into a methanol:dichloromethane mixture (9:1).

Reversed phase chromatographic separation of the samples was achieved on a Waters Acquity BEH C18 column with a Dionex Ultimate 3000 RS UHPLC. HGLs were detected by a maXis quadrupole time-of-flight mass spectrometer (Bruker Daltonics) in positive ionization mode and in a mass-to-charge (*m/z*) range from 150 to 2000; MS^2^ scans were obtained in data-dependent mode. HGLs were identified by monitoring exact masses of possible parent ions (present as either H^+^, NH_4_^+^ or Na^+^ adducts) in combination with characteristic fragmentation patterns as outlined by Bauersachs et al. [39] and Wörmer et al. [5]. By comparing peak areas of HGLs to the peak area of a known amount of an internal standard (1,2-diheneicosanoyl-sn-glycero-3-phosphocholine, C_21_-PC) added to the sample before injection onto the HPLC-MS system, their concentration could be semi-quantitatively assessed. A response factor of 1 compared to C_21_-PC was assumed for all HGLs.

**Nitrogenase activity measurement.** Nitrogenase activity was measured by acetylene reduction in a method that has been established for cyanobacteria [40]. Briefly, cultures were incubated in the presence of acetylene for several hours at normal culturing conditions in flasks closed by gas tight caps. To generate anoxic conditions, before incubation with acetylene, a solution of DCMU (10 μM dissolved in methanol) was added to cells, and then closed flasks were filled with argon and incubated for one hour. After incubation with acetylene, 1 mL of the gaseous phase was taken from each flask, and the amount of ethylene produced was measured by gas chromatography.

**Study of interactions between HgdD and HgdB.** Gene fragments of *hgdD* and *hgdB*, encoding soluble parts of the proteins, were used for the experiment. In HgdD, a fragment of the gene lacking the periplasmic N-terminus part (amino acids 1 to 287), which is not found in the sequence of the TolC from *E. coli*, was cloned into pET42a vector (Novagen, Merck) so that the protein would bear the GST-tag at the N-terminus of the peptide. The membrane domains of the HgdD (amino acids 365 to 418 and 586 to 625) were substituted by glycine-serine linkers using PCR. In case of HgdB, the membrane helix sequence (amino acids 13 to 42) was replaced by a glycine-serine linker as well, and the construct was cloned into the pASK-IBA3 vector (IBA Lifesciences), so that the protein would bear the Strep-tag at the C-terminus (also see the scheme in Figure 7A; the primers used are listed in Table S4). The proteins were overexpressed in *E. coli* Lemo21 (DE3) strain and purified using affinity chromatography according to standard protocols provided by GE Life Sciences and IBA Lifesciences. The pure proteins (4 or 10 μM) were mixed with each other and the Strep-Tactin-coated magnetic beads (IBA Lifesciences, Göttingen; #2-4090-002) to perform a pull-down experiment. The mixture was incubated at 28 °C for one or two hours in binding buffer (20 mM Tris, 200 mM NaCl, pH 7.5) with occasional mixing and then three wash steps with the same buffer were performed. Bound proteins were eluted by 50 mM desthiobiotin-containing elution buffer (IBA Lifesciences). SDS-gel electrophoresis was done with all pull-down fractions.

**Measurement of ethidium bromide accumulation.** Measurement of accumulation of ethidium bromide (EtBr) in the cells was done according to the previously described method [41], except for minor modifications. The strains were spotted onto BG11-agar plates containing 0.5 μg/mL EtBr. Each drop contained 0.5 μg chlorophyll *a*. The plates were incubated at low light (4 μmol photons m^−2^ s^−1^) at 28 °C for 16 h. Then the fluorescence of colonies, which accumulated EtBr, was captured using an UV-transilluminator, and the relative intensity of the fluorescence was quantified and analyzed by GelQuantNET and GraphPad Prism programs.

## 3. Results

### 3.1. HgdBC Is Differentially Expressed in Heterocysts and Localizes at the Membrane

Transposon mutant FQ777 was shown to be impaired in diazotrophic growth and was not able to form normal laminated layers [22]. The transposon tagged gene encodes a putative membrane fusion protein HgdB, and localizes next to a gene encoding an inner membrane factor, HgdC (Figure 1A). To investigate the function of *hgdB* and *hgdC* in more detail, we followed the gene expression of *hgdB* and *hgdC* during nitrogen starvation and localized the putative ABC transporter by a fusion of GFP to HgdC.

Expression of the *hgdB* and *hgdC* genes was studied using the semi-quantitative RT-PCR technique. Cultures, grown in presence of NH_4_^+^ were transferred by stepdown to NH_4_^+^-free media in liquid cultures. Heterocysts could be detected after 24 h under these conditions. The *hgdB*-transcripts were present at a low level in NH_4_^+^-grown cultures but increased clearly between 24 h and 48 h and remained at high levels afterwards (Figure 1B). A similar expression pattern was observed for the *hgdC* gene, whose expression was also significantly up-regulated after 48 h after nitrogen removal from the medium (Figure S3). This expression profile showed that *hgdB* is up-regulated later than the *devB* gene (HGL exporter component), which is transiently expressed between 15 h and 24 h after the removal of the combined nitrogen source (Figure 1B).

A possible NtcA binding site was detected starting at the position of -629 base pairs upstream of the *hgdB* gene by *in silico* search. The sequence of this site is 5′-GTAGGCATAGTTAC-3′, which perfectly fits to the consensus sequence 5′-GTA-N_8_-TAC-3′ of NtcA binding sites in cyanobacteria [42,43]. NtcA, as the global transcription factor controlling the expression of different heterocyst genes including *devBCA* [44], could also be responsible for the differential expression of the *hgdB* gene.

The heterocyst-specific expression of the HgdBC putative transporter complex was confirmed by localization studies using in-genome translational fusions of *hgdC*, encoding the permease domain, to the *sfGFP* gene (superfolder green fluorescence protein [31])

After single recombination of plasmid pIM706, bearing a fragment of the *hgdC* gene that corresponded to the C-terminus of the protein fused in frame to *sfGFP* by a glycine-serine linker (Figure 2A), green fluorescence was observed in the periphery of the heterocyst after 48 h of nitrogen stepdown. The GFP-fused HgdC protein was homogenously distributed all over the cytoplasmic membrane without any preference to the polar or lateral side, with a paucity at the polar end, where the heterocyst is connected by a small septum to an adjacent vegetative cell (Figure 2B,C). This localization pattern corresponds with *in silico* predicted membrane localization of HgdC protein and the absence of the heterocyst envelope inside the polar septum.

### 3.2. The HgdB Mutant Is Impaired in Diazotrophic Growth

To investigate the function of the putative HgdBC transporter in more detail, we created a site-directed mutant. The *hgdB* gene was insertionally inactivated after homologous single recombination with the plasmid pIM652 (for the scheme of the mutational approach and the verification of mutant segregation see Figure S2). The fully segregated mutant strain SR652 (*hgdB*::pIM652) grew similar to the wild-type strain on medium supplied with a nitrogen source, but was not able to grow on nitrogen-free medium (Figure 1C), confirming the Fox^−^ phenotype of the transposon mutant FQ777 [22].

There were no visible differences in filament morphology between the mutant and the wild type using light microscopy. Filaments of the mutant did not show any aberrations and were of the same length. Cells were of the same size as wild-type vegetative cells.

### 3.3. The HgdB-Mutant Forms an Aberrant Heterocyst Cell Envelope

**Figure 3:**
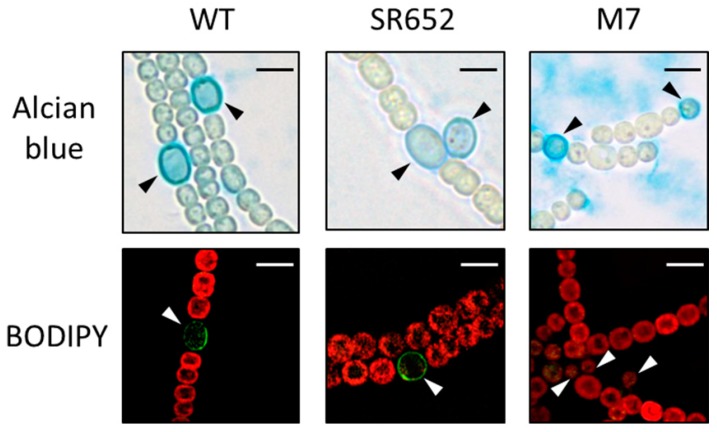
Micrographs of the SR652 filaments in comparison to the WT and DevA-deficient mutant M7. Alcian blue stains exo-polysaccharides in blue; the green fluorescent dye BODIPY binds to neutral lipids. The red color indicates the cyanobacterial autofluorescence coming from the photosynthetic pigments. Heterocysts are indicated by triangles. Both staining procedures were performed after three days of nitrogen starvation. Bars, 5 μm.

Upon nitrogen stepdown the *hgdB* mutant SR652 formed heterocysts, which were clearly visible by light microscopy after 3 days of induction in nitrogen-free medium. The presence of the hep layer was confirmed by staining the polysaccharide envelope with Alcian blue (Figure 3, upper panel) [34].

The envelope glycolipids were stained by the fluorescent dye BODIPY (Figure 3, lower panel), which specifically stains neutral lipids (hence the hgl layer) and in some cases also intracellular lipid droplets [32]. The heterocyst envelope of the wild type and mutant strain SR652 had clearly adopted the green fluorescence. In contrast, the *devA* mutant M7 [21], which is not able to transport the glycolipids to the envelope, was not stained by BODIPY, demonstrating the lack of the laminated layer (Figure 3, lower panel).

Also, staining of the peptidoglycan with fluorescent vancomycin [33] did not show any differences between the wild-type and the SR652 strain (Figure S4). The dye predominantly bound to the septal peptidoglycan in vegetative cells but was also present all over the periphery of heterocysts.

**Figure 4:**
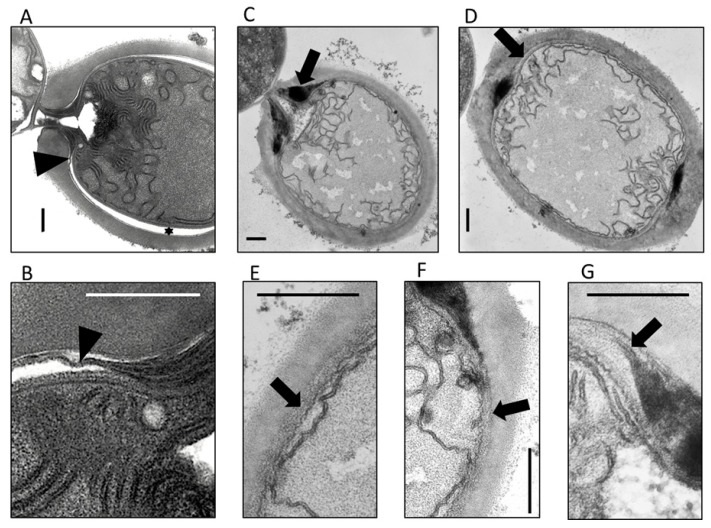
The ultrastructure of heterocysts from the mutant SR652 in comparison to the wild type of *Anabaena*. (**A**,**B**) Heterocyst of the wild type. Triangles indicate the laminated hgl layer, the star points to a preparation artifact due to dehydration during sample preparation. (**C**–**G**) SR652 mutant heterocysts. Arrows show an anomalous layer between the cytoplasmic membrane and the polysaccharide layer, which sometimes appears within the hgl layer (**G**). Bars, 0.4 μm.

To investigate the non-functional heterocysts in more detail, the ultrastructure of ultrathin sections was analyzed. The vegetative cells did not present any differences between the wild type and the SR652 mutant (not shown), and the heterocysts of SR652 displayed the outer hep layer like the wild-type heterocysts (Figure 4). However, differences were obvious in the laminated layer. Compared to the wild type, it was much thicker around the so-called polar neck at each end of the heterocyst (see also figures in [18,20] and Figure 4C,G). A less-structured layer was evident below the hep layer, which resemble neither the hgl nor the hep layer (Figure 4C–G). In contrast to the wild type, where a thin electron dense layer went all around the heterocyst between the cell wall and the hep layer, the transition of the massive hgl deposition from the neck area to the heterocyst circuit was not evident in the mutant heterocyst. Instead, the laminated layer transformed to this abnormal extra layer, which then encompassed the entire heterocyst. In some sites, the extra layer appeared between two darker stained laminated layers, similar to previous observations (see Figure 4G and Figure 2 in [22]).

**Figure 5:**
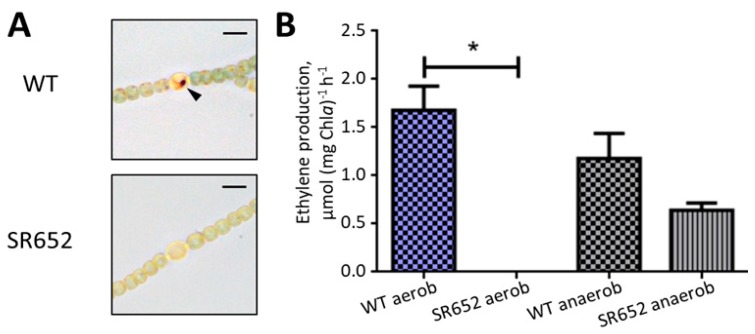
Oxygen protection of nitrogenase in the mutant SR652. (**A**) Triphenyl tetrazolium chloride (TTC) staining of WT and SR652 mutant filaments. TTC reduces to dark formazan crystals (black triangle) under micro-oxic and reducing conditions, which are necessary for the proper activity of the nitrogenase complex. Bars, 5 μm. (**B**) Assessment of the nitrogenase activity in the mutant SR652 and the wild type (WT) under aerobic and anoxic conditions using the acetylene reduction assay. Data are representative of two independent experiments. The histogram shows the mean values ± SD of two experimental replicates. Student’s t test *P* value is *P* < 0.02 (*).

In the case of aberrant envelope formation, heterocysts are not able to produce a micro-oxic environment suitable for nitrogenase. To check the micro-oxic situation in the heterocysts of strain SR652, the filaments were incubated with TTC, which forms dark-red or brown precipitates under low oxygen (hence in heterocysts of the wild type) [35]. No such precipitates were detected in the mutant heterocysts (Figure 5A). To investigate whether the mutant was unable to fix nitrogen, the nitrogenase activity was measured by the acetylene reduction assay [40]. Only under anoxic conditions the nitrogenase activity of the mutant was detectable, although it was lower than in the wild type. There was no activity under normal aerobic conditions (Figure 5B). This is a clear indication that heterocysts of the mutant cannot provide the micro-oxic environment for the nitrogenase, which can be active only under anoxic conditions in the mutant. This phenotype is typical for mutants defective in the heterocyst envelope [45].

### 3.4. The Glycolipid Composition Is Affected in the HgdB-Mutant

**Figure 6:**
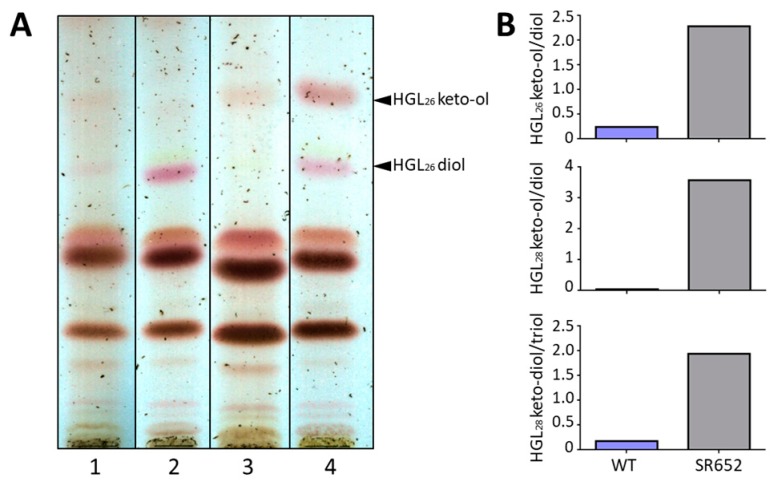
Analysis of heterocyst-specific glycolipids from whole cell extracts of the mutant SR652 and the WT. (**A**) Thin-layer chromatography of lipid extracts from liquid cultures before and 3 days after nitrogen stepdown; 1—wild type with nitrate, 2—wild type without nitrate, 3—SR652 with nitrate, 4—SR652 without nitrate. Black triangles indicate two major forms of heterocyst-specific glycolipids (HGLs). Shown is a representative thin layer chromatogram of five independent experiments; (**B**) Ratios of different keto-variants of HGLs to their corresponding hydroxy forms.

Since the presence of the hgl layer in the heterocyst envelope was demonstrated microscopically, we analyzed the lipid composition in methanolic extracts of the SR652 mutant compared to the wild type by TLC and HPLC-MS (Figure 6). As expected, in NO_3_^−^ grown cultures almost no HGLs were present in any of the strains. However, after nitrogen removal for three days, both heterocyst-specific glycolipids appeared in higher amounts, displaying an obvious difference in their ratio between the mutant and the wild type. In the mutant, the upper band corresponding to the keto-ol form of HGL (1-α-glucosyl-3-keto-25-hexacosanol, HGL_26_ keto-ol) [32] was noticeably stronger than in the wild type, whereas the band containing the diol variant (1-α-glucosyl-3,25-hexacosanediol, HGL_26_ diol) [32] was weaker (Figure 6A). A HPLC-MS analysis of extracted lipids from the mutant strain revealed the presence of six heterocyst glycolipids, with the C_26_ aglycone-containing variants forming the largest group. The clear difference in the ratio between the HGL_26_ keto-ol and diol forms in the wild type and the mutant in whole lipid extracts was confirmed (Figure 6B). Interestingly, a similar change in the ratio was shown in the mutant for minor HGLs with a C_28_ carbon chain: for HGL_28_ keto-ol and diol variants, and HGL_28_ keto-diol and triol variants. Hence, the ratio between the different lipid forms was affected in the mutant resulting in an aberrant hgl layer.

### 3.5. HgdB Interacts with HgdD in Vitro

**Figure 7:**
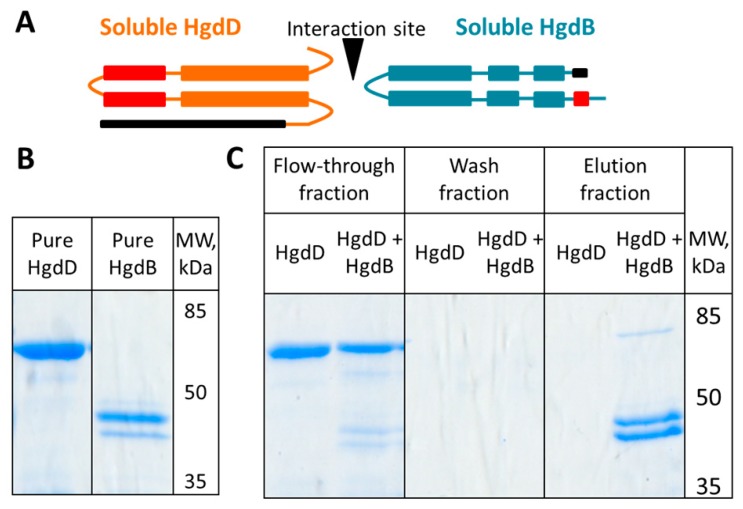
Pull-down assay showing an interaction between purified soluble HgdD (TolC) and HgdB (All5347). (**A**) Scheme of the soluble proteins of HgdB and HgdD used for the assay. Tags (GST for HgdD and Strep for HgdB) are shown in black, membrane domains substituted by glycine-serine linkers are depicted in red; (**B**) SDS-PAGE of the purified HgdD and HgdB fragments; (**C**) SDS-PAGE of pull-down fractions. MW—molecular weight.

Assuming that HgdB is part of a T1SS-like transporter system with HgdD (TolC), we studied possible interactions between these two proteins *in vitro*. Soluble parts of HgdB, with a Strep-tag and TolC with a GST-tag (Figure 7A) were expressed in *E. coli* from plasmids pIM701 and pIM689, respectively. The soluble domains were selected according to their predicted structure and protein-protein interaction sites [17]. The domains were purified using affinity chromatography (Figure 7B). The HgdD peptide bound to the Strep-Tactin-coated magnetic beads only when applied together with the HgdB-Strep-tag peptide, which clearly showed the interaction between these proteins (Figure 7C).

In order to test whether the putative transporter HgdBC-HgdD plays a role in multidrug resistance of *Anabaena*, we measured the retention of EtBr in mutant SR652 in comparison to TolC- and DevA-deficient mutants and the wild type. Only the TolC-deficient mutant cells accumulated EtBr, while all other strains did not (Figure S5). This observation is consistent with a previous similar study of a mutant in the *hgdC* gene, which did not show a role of the *hgdB-hgdC* gene cluster in drug efflux [46].

## 4. Discussion

The formation of the heterocyst hgl layer, the actual barrier for oxygen diffusion into the mature heterocyst, is not yet fully understood. Numerous genes have been identified that are involved in HGL synthesis and deposition (see reviews by [2,47]). Previously, the group of Peter Wolk identified a gene island that comprises several genes involved in hgl layer formation. Transposon mutants in those genes showed the Fox^-^ phenotype [22], moreover, the inability of a mutant in the *hgdC* gene to grow diazotrophically was confirmed by Hahn and colleagues [46]. The function of clustered genes *hgdB* and *hgdC* was reexamined in more detail in this work. By encoding an MFP and an IMF similar to DevB and DevC, HgdB and HgdC may form another T1SS-like transporter, which uses an ATPase encoded by a gene outside the cluster. In many cases, single genes encoding ABC transporters’ subunits (a permease and an ATPase) are found distributed over a genome [48]. The expression of *hgdB* (as well as *hgdC*) starts during a phase of heterocyst maturation, which was noticeably later than the induction of the homologous *devB* gene. Since DevB is involved in HGL export, the HgdBC system may be involved in a process following the export of the glycolipids by DevBCA. A putative NtcA binding site [42,43] upstream of the *hgdB* gene suggests its expression being NtcA-dependent. We could not find such a site upstream of *hgdC* (except the sequence 5′-GTAAAGCTTTAC-3′, which is similar, but not identical to the consensus 5′-GTA-N_8_-TAC-3′). This could indicate a different mechanism of regulation for the *hgdC* and *hgdB* genes in the cluster. In contrast, the *devBCA* genes are co-transcribed under control of NtcA [44]. From the complementation study by Fan et al. [22] it can be concluded that the genes *hgdB*, *hgdC*, and *hgdA* are independently transcribed, each being a Fox^−^ gene.

To localize the putative secretion system involving HgdB and HgdC, sfGFP was fused to the C-terminal end of the HgdC protein and transferred to *Anabaena*. As expected, the protein appeared in mature heterocysts, in line with the results from RT-PCR (Figure 1C and Figure S3). Fusion protein HgdC-sfGFP was localized throughout the periphery of the heterocyst (Figure 2B), presumably residing in the cytoplasmic membrane, as expected for an IMF protein. Interestingly, the protein was not present at the polar neck regions (Figure 2B,C). This indicates that the membrane in the septa between heterocysts and vegetative cells lacks the HgdC protein, indicating an unknown sorting mechanism, which directs a membrane complex to the membrane, excluding the septal area. Assuming that HgdBC is involved in hgl layer formation, this result is in line with the absence of the heterocyst envelope at the septa. Furthermore, HgdB interacts with the outer membrane protein HgdD, which does not reside in the septum, where the outer membrane is not present [49].

By insertional inactivation of the *hgdB* gene in strain SR652, we confirmed the importance of HgdB for heterocyst function and normal diazotrophic growth of *Anabaena* sp. PCC 7120.

Even though the mutant was not able to grow on N_2_ as nitrogen source, heterocysts formed both layers (Figure 3). Also the peptidoglycan layer did not show any abnormality (Figure S4). Interestingly, in wild-type and mutant heterocysts, fluorescent vancomycin labeled the entire cell wall, showing that during heterocyst maturation peptidoglycan turnover or modification is taking place. In contrast, vegetative cell growth originates from the septum, where obviously new peptidoglycans are incorporated. A similar observation was described recently by using a fluorescent analog of the peptidoglycan precursor D-Ala [50].

The reason for the Fox^-^ phenotype of SR652 lies in abnormal HGL deposition as previously described for the transposon mutant FQ777 [22]. The hgl layer is the essential barrier to the O_2_ penetration into heterocysts [47,51]. Mutants with aberrant heterocyst envelope layers lose the ability to fix N_2_ in the air; however, when incubated under anoxic conditions, nitrogenase activity develops, as in mutant SR652. This is another indication of an aberrant envelope structure, as evident in electron micrographs of ultrathin sections. There, an additional unstructured layer between the outer membrane and the hep layer appeared, as well as several other structural differences (e.g., cytoplasmic and thylakoid membranes showed a somewhat wavy structure, and the hgl layer was thicker at the polar neck) (Figure 4). The electron-dense thin hgl layer, which enwraps the entire heterocyst, was not visible. These findings principally match those of the *hgdB* mutant FQ777 [22], with some differences. In the previous study, the additional layer was identified in electron micrographs as the hep layer, which is aberrantly deposited under the hgl layer, and was caused by aberrant timing of deposition of envelope polysaccharides before and after HGL deposition. In that study, the polar neck was not shown for FQ777, but the *hgdC* mutant FQ632 showed a similar dense deposition of HGLs around the polar neck, like our *hgdB* mutant SR652. This indicates that HgdC and HgdB probably fulfill the same function and apparently form together an ABC transporter involved in correct HGL deposition. 

Similar to other heterocyst envelope mutants, nitrogenase was active in the SR652 mutant only under anoxic conditions (Figure 5). Somewhat lower rates of activity in the mutant compared to the wild type can be explained by the nitrogen starvation of the strains before the measurement. In addition to the nitrogenase activity measurements, TTC staining showed that the mutant does not have micro-oxic conditions inside heterocysts. All these findings support the idea that the mutant heterocysts are surrounded by a nonfunctional gas-permeable cell envelope and therefore cannot provide the micro-oxic compartment essential for nitrogenase function.

The chemical analysis by TLC and HPLC-MS of the HGLs allowed us to suggest that the composition of the hgl layer is aberrant in terms of the ratio of the oxidized (keto-ol or keto-diol) to the reduced (diol or triol) form of the HGLs (Figure 6). While different HGL species have been described in several studies before, the reduced forms are typically dominant, and ratios such as the ones observed in the present study are not reached [6,7,9,10,11,12,52].

Interestingly, the ratio of oxidized and reduced HGL species has been used as an indicator for growth temperature of HGL-producing organisms [5,53], indicating the relative abundance of diols compared to keto-ols with increasing temperature. Higher temperatures might lead to a rise in permeability of the hgl layer, which would be compensated by increasing the diol amount. This supports the hypothesis that the correct diol to keto-ol ratio is mandatory for proper permeability of the envelope. We have presented here a new finding implying that both aglycones and their specific, tightly regulated quantitative ratio to each other is essential. To our knowledge, this is the first experimental evidence of the importance of the correct ratio of both derivatives of the HGLs for the correct assembly and function of the hgl layer as a gas barrier.

As shown in a previous study by Valladares et al. [54], a Fox^-^ mutant in heterocyst-specific terminal respiratory oxidases also showed in lipid extracts of isolated heterocysts an aberrant ratio between the two major forms of HGLs in TLC, but the laminated layer looked normal in electron micrographs. In contrast to the *hgdB* mutant, the *prpJ* (*all1731*) and the protein-kinase double mutant D4.3 did not synthesize the major and the minor HGL, respectively, and there was no deposition of HGLs demonstrated [47,55,56]. This implicates that the presence of both HGLs is necessary for HGL deposition. Both mutants were not able to fix N_2_ under oxic conditions, confirming that both HGLs are essential for heterocyst function.

The changes in HGL composition in the *hgdB* mutant could be linked to the possible functions of the product of the *hgdA* (*all5345*) gene, which is independently transcribed from upstream gene *hgdB* [22]. *In silico* analysis predicts its possible epimerase or short chain dehydrogenase/reductase (SDR) families’ affiliation. Therefore, it might either convert the keto-ol HGL form to the diol form or perform some epimerization reaction to produce glycosyl epimers as specific substrates of glycosyltransferases known to be involved in HGL synthesis [47,57].

The aberrant ratio between two main HGL forms might lead to an improper packaging of the HGLs in the laminated hgl layer around the heterocysts, resulting in the anomalous thick envelope between the hep layer and the outer membrane of the heterocyst (see above and Figure 4). The incorrect deposition of the hgl layer probably causes its permeability for gases and does not allow protection of the nitrogenase.

**Figure 8:**
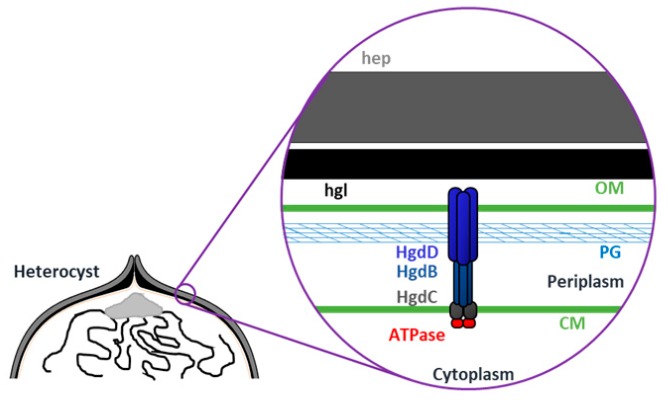
Model of the putative TolC-HgdBC in the cell wall of heterocysts. The TolC-HgdBC is present in the whole perimeter of the heterocyst except the polar neck and perhaps exports HGLs, a specific protein, or another factor, crucial for correct hgl layer formation. Hep—polysaccharide layer, hgl—glycolipid layer, OM—outer membrane, PG—peptidoglycan, CM—cytoplasmic membrane.

Despite the lack of an evident transporter function, HgdB (All5347) could interact with HgdD (TolC) *in vitro* (Figure 7). This observation supports the assumption that HgdB forms, together with HgdD, a transporter of the T1SS-like type similar to DevBCA-HgdD (Figure 8). Furthermore, the promiscuity of the HgdD (TolC) protein in cyanobacteria has already been shown [16,17,41,58,59]. So far, an ATPase subunit is missing in this transporter. However, this function could be fulfilled by DevA, which is specifically expressed in heterocysts and before the *hgdBC* transcription, or another ATPase. In the genome of *Anabaena*, 91 genes encoding ATPase subunits are present [15].

In this work, we investigated the role of the genes *hgdB* and *hgdC*, homologous to *devB* and *devC*, and their products in heterocyst development in *Anabaena* sp. PCC 7120. We showed their involvement in this process and, in particular for *hgdB*, their participation in formation of the proper hgl layer in the heterocyst cell envelope. It is worth mentioning that other *devBCA* homologues are not as highly up-regulated under nitrogen starvation as *hgdBC* and *devBCA* [60], and probably fulfill functions unrelated to diazotrophic growth. However, other types of transporters participating in heterocyst formation and diazotrophic growth in *Anabaena* have been described [61,62,63]. Probably, the functions of the encoded proteins are not directly linked with the transfer of HGLs to the envelope, but rather with their assembly and modification pathways. Of great importance is the putative reductase HgdA (All5345), which may be a novel enzyme with a significant role in hgl layer formation (manuscript in preparation). Future work should focus on investigation of functions of this particular enzyme and its partners.

## Supplementary Materials

The following are available online at http://www.mdpi.com/2075-1729/8/3/26/s1, Figure S1: Alignment of amino acid sequences of HgdB with DevB (A) and HgdC with DevC (B); Figure S2: Segregation of the mutant SR652; Figure S3: Time-dependent expression analysis of *hgdC* and *hgdB* genes in the wild type during nitrogen starvation studied by RT-PCR; Figure S4: Vancomycin-FL staining of WT and the SR652 mutant filaments; Figure S5: Retention of ethidium bromide by the SR652 mutant in comparison to the WT, the TolC-deficient mutant DR181, and the DevA-deficient mutant M7; Table S1: *Anabaena* sp. PCC 7120 strains used in the work; Table S2: *E. coli* strains used in the work; Table S3: Plasmids used in the work Table S4: Oligonucleotides used in the work.
