# Supplementary material for: The ABC Transporter Components HgdB and HgdC are Important for Glycolipid Layer Composition and Function of Heterocysts in Anabaena sp. PCC 7120
Source: Life (Basel). 2018 Jul 2;8(3):26. doi: 10.3390/life8030026 (PMC6161253; doi:10.3390/life8030026)
Supplement: Supplementary file 1 [file life-08-00026-s001.pdf]

Supplementary data.

A

```
      1       10        20        30        40        50        60        70        80        90       100       110       120      130
HgdB  MAVNKERQLFIKPQGRWRLILAASLTLAAGLISLYSLSSNRFSAPVKTPANTPKAAPVRVAVTALGRLQPQGQITTLSAP--SSTNGIRVDRLLVKEGDEVQAGQVLAYLEDYSRATAALEQALDKLQI
DevB MSRVTEERRSREQPLDLEQPKIWWGLAVALPVAVAAGLLATAKFEQLKRLGPSVPVKPVITSVSAVGRLEPQGEVIKLSAPVGGMQSASRVKQLFVKEGERVRKGQVIAILDNHDTQLAAVEEAKAKLLE

     131     140       150       160       170       180       190       200       210       220       230       240       250      260
HgdB AKIKLAQVKAGAKPGDIDAQKATVARLESQLKGEVATQQA------------------------------------TINRIKAELNNAQTENDRYQKLFKEGAVSAS-----------------------
DevB SRANLAQVRVGS-PRDIQAQTAVIARLQAQLVGEMGAQQASITRIASQLSGEKVAQQALVNRLEAELVGQQDSLRATLNRIRAEQRNAQVDAGRYDFLYREGAISQQERDRRRLTATTANQQVIESQAAL

     261     270       280       290       300       310       320       330       340       350       360       370       380      390
HgdB ----------VFDSKALQLKTV---EQQLTEAKASLSRSQNTLADQIKEAKATLSSVKEVRAVDVDLAQMEVRSAVTAVKQAKADQELTYIKAPIDGRILKTHAKTGEVIPSSGFADIGKTSQMYVIAEV
DevB RQALATLRQQVAEARANQMKTLASLQQQLIEARVTRDKTITTLQRQIDEEKARLKRLVEVSPTDVQMAQAQVSNAIANVRRAEAELKLSYIQAPSAGEILKIYSKSGEAITADGIAEMGETEQMMIIAEV

     391     400       410       420       430       440       450       460       470   476
HgdB YQTDIQKVRVGQKATITST--AFSGKLQGTVREIGWQVDKQSIFSLNPRSDTDRRIIEVKVSIDNPADSERVGRLTNLQVDVAIQI
DevB PEDSIGRVRIGQTVTANSDNGAFSGEIRGTVTEIGRKIGKKDVLNTDPAADVDARVVEVKIAL-SPEDSQKVSGLTYAKVVVDINK
```

B

```
     1       10        20        30        40        50        60        70        80        90       100       110       120      130
HgdC MNFK-IPLAWLQLVQQKIRFLIAVAGIAFIVLLMFVQLGFQDALYSSATAVHQNLRGDLFLVSSQYKSLTASQSFSRTRLYQALGFDGVESVSPMYLQFAKLKNPETGEKYSIYVIGFDPGQPVMNIPEV
DevC MNRKRIPLSWLQLTREKTRLAVALAGIAFADILMFMQIGFRDALYYSNVRLHTSLQGDIVLLNKQSNAVLAMKPFSQRRLYKSLELPSVQSVHPIYLDYSIWKNPYTGLTRSILVFGINPETNIFNLAGV

     131     140       150       160       170       180       190       200       210       220       230       240       250      260
HgdC EENLDKLKIPDVMLFDRDSRPEFGPIAANFNKGDTEQTIEIFPFDAPIGYKVRVGGLFSLGPSFGVDGNLIVSDSTFLRINPNTRPAEKIDIGAITLKPGADQEKIVANLEANLPNDVRVFTRKGFIDFE
DevC QENLDKLKLPDTVLFDRSSRPEYGPIAKNFEQGQSISA-------EVRRRRINVVGLFTLGASFGADGNLVTSDVNFLRLFPLRRQG-LIDIGLIRLKPGVNPNTAVQELRSYLPDDINVLTKQEFIDFE

     261     270       280       290       300       310       320       330       340       350       360       370       380      390
HgdC KQYWSVRTPIGFILNIMLTMASVVGVVIVYQILYSNIATQFIAYATLKAIGYPNIYLLNVVFQQALILALLAYIPGFLFAVGLYDFAMTATKLPIVMTANNALIVFVSVVLMCITSGALAINKLRSADPA
DevC RNYWASSTAIGFIFTLGTIMGFIVGTVIVYQILYTEVADHLSEYATLKAIGYTQKYLLGVILQEALLLACLGYLPGWIFAIILYKTARQATLLPVFMSYDRAITVLILTMIMCFVSGSIAVRKLRSADPA

     391
HgdC DIF
DevC DIF
```

Figure S1. **Alignment of amino acid sequences of HgdB with DevB (A) and HgdC with DevC (B)**.
Alignment was performed using Multalin software [26]. Red color indicates highly similar areas, blue and black colors show low similar areas.

Supplementary data.

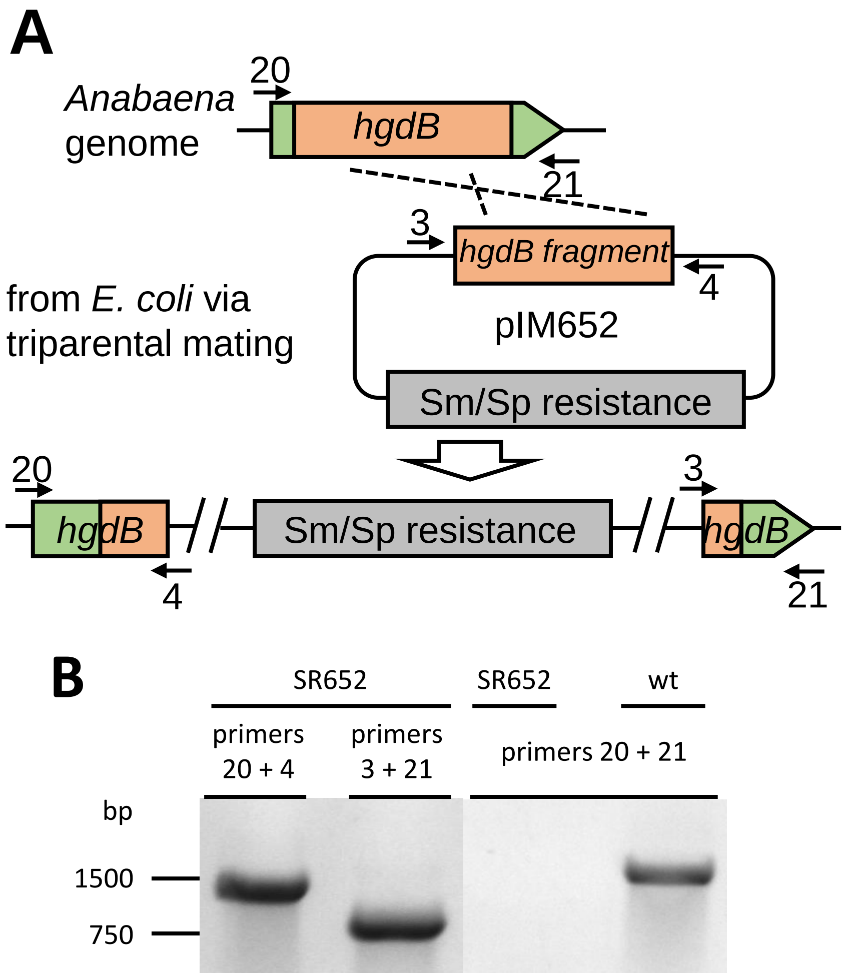

Figure S2. **Segregation of the mutant SR652.**
A, Scheme of homologous recombination to obtain the single recombinant SR652 mutant. Arrows with numbers indicate primers (Tab. S1) used for the genotypic analysis of the mutant; **B**, Genotypic analysis of the mutant; SR652 - mutant colony was used as a template for PCR, wt – wild type colony was used as the template. Numbers correspond to those depicted on **A**.

Supplementary data.

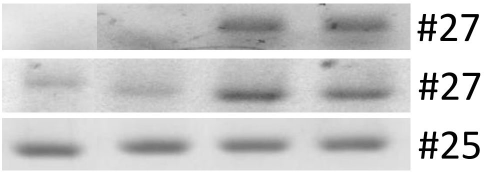

Figure S3. **Time-dependend expression analysis of *hgdC* and *hgdB* genes in the WT culture during nitrogen starvation by RT-PCR**. *rnpB* corresponds to ribonuclease B, which was used as a positive control. Numbers at the right side of the table are numbers of PCR cycles.

Supplementary data.

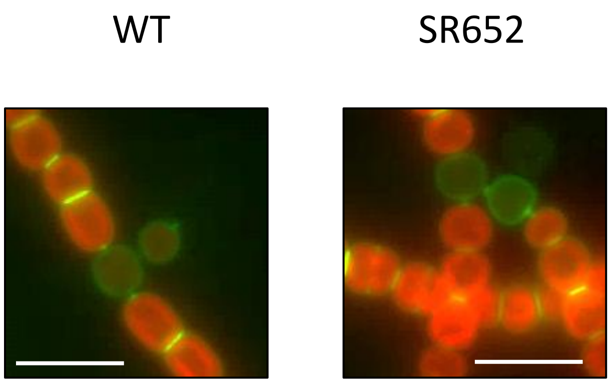

Figure S4. **Vancomycin-FL staining of WT and SR652 mutant filaments.**
Vancomycin-FL (green fluorescence) is a fluorescent stain that binds to the peptidoglycan. Red color corresponds to the autofluorescence of cells. Bars are 5 µm long.

Supplementary data.

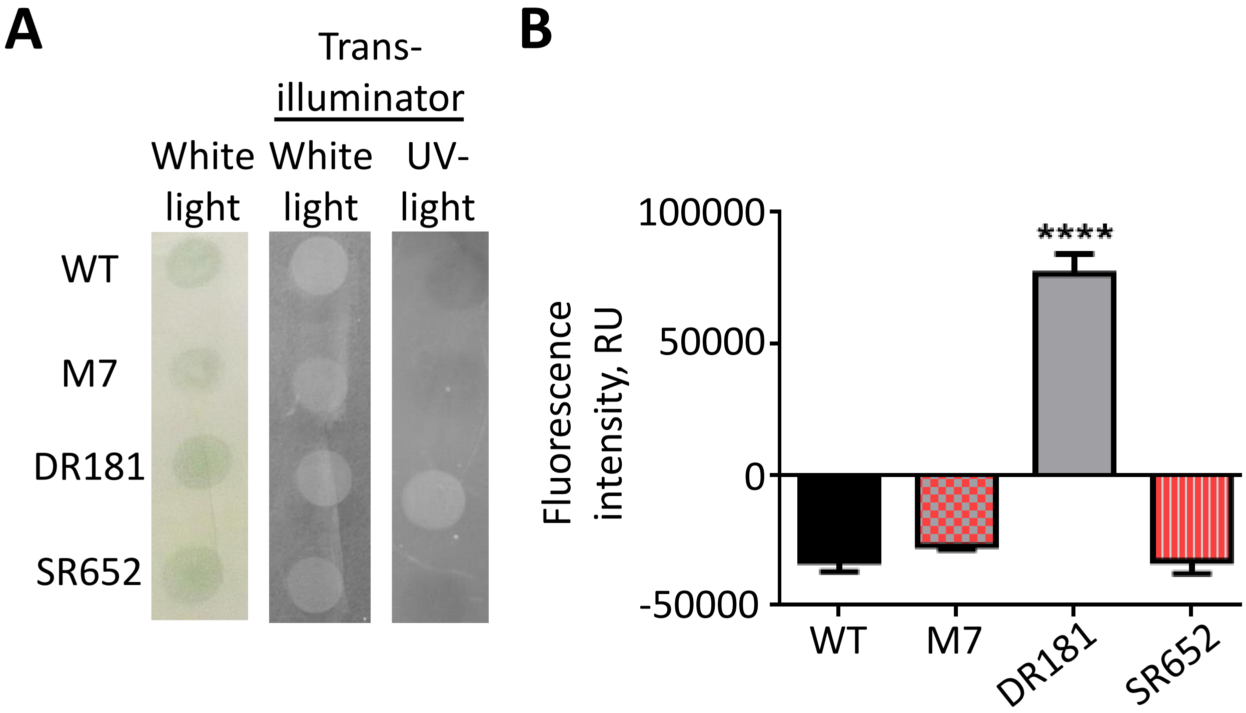

Figure S5. **Retention of ethidium bromide by the SR652 mutant in comparison to the WT, the TolC-defficient mutant DR181, and the DevA-defficient mutant M7.**

A, Behavior of *Anabaena* WT and mutant cultures on the solid BG11 medium containing 0.5 µg/ml Ethidium bromide after 16 hours growth starting at 0.1 µg of chlorophyll a; **B**, evaluation of EtBr retention by calculating the relative intensity (RU – relative units) of EtBr fluorescence under UV-light in *Anabaena* colonies. The histogram shows the mean values ± SD of two independent experiments. Student's *t* test *P* value is < 0.0001 (****).

Supplementary data.

Table S1. *Anabaena* sp. PCC 7120 strains used in the work.

Number	Name	Reference
1	*Anabaena* sp. PCC 7120 wild type	[27]
2	SR652	This work
3	SR706	This work
4	M7 (DevA-defficient mutant)	[21]
5	DR181 (TolC-defficient mutant)	[18]

Table S2. *E. coli* strains used in the work.

Number	Name	Reference
1	NEB10	New England Biolabs
2	Top10	Invitrogen
3	HB101 (pRL528)	[28]
4	J53 (RP4)	[28]
5	Lemo BL21 (λDE3)	Novagen/Merck

Table S3. Plasmids used in the work.

Number	Name	Reference
1	pIM652	This work
2	pIM706	This work
3	pRL277	[57]
4	pET42a	Novagen/Merck
5	pASK-IBA3	IBA Lifesciences
6	pIM701	This work
7	pIM689	This work

References:

57. Black, T. A.; Cai, Y.; Wolk, C. P. Spatial expression and autoregulation of *hetR*, a gene involved in the control of heterocyst development in *Anabaena*. *Mol. Microbiol.* **1993**, *9*, 77-84, doi:10.1111/j.1365-2958.1993.tb01670.x.

Supplementary data.

Table S4. Oligonucleotides used in the work. All primers were purchased from Sigma-Aldrich.

Number	Primer	Purpose
1	5’ATAATAAGCGGATGAATGGCAGAAATTCGATATCTAGATCTTTCCGCACCAGTCAAGACC3’	Cloning for *hgdB* mutant generation
2	5’CCGGCATGTCCCCCTGGCGGACGGGAAGTATCCAGCTCGAGAGGGAGGCTTTGGCTTCTG3’	Cloning for *hgdB* mutant generation
3	5’TTAAACGCCTGGTGCTACGC3’	pRL277 fw primer
4	5’GGCCTCTTCATCGGGAATGC3’	pRL277 rw primer
5	5’CTGAATAAGTGATAATAAGCGGATGAATGGCAGAAATTCGATATCTAGATCACAATGGCTTCTGTTGTG3’	Cloning for *hgdC-sfgfp* fusion
6	5’TACTACACCTGTAAATAATTCTTCACCTTTTGAAGAGCCTCCTCCACCAAAAATATCAGCCGGGTCAGC3’	Cloning for *hgdC-sfgfp* fusion
7	5’CTTCGTTCTGCTGACCCGGCTGATATTTTTGGTGGAGGAGGCTCTTCAAAAGGTGAAGAATTATTTACAG3’	Cloning for *hgdC-sfgfp* fusion
8	5’CATCGCCGGCATGTCCCCCTGGCGGACGGGAAGTATCCAGCTCGATTATTTATATAATTCATCCATACCATGTGTA ATA3’	Cloning for *hgdC-sfgfp* fusion
9	5’GGCAAAAAATGGGAGACCGCGGTCCCGAATTCGAGCTCGGTACCCGGGGATCCCATGGCAGTAAATAAAGAAC 3’	Cloning and engineering of *hgdB* gene for expression of the soluble part
10	5’TCGAACTGCGGGTGGCTCCAAGCGCTGAGACCATGGTCCCCCTGCAGGTCGACCTCGATAATTTGAATCGCTACA TC3’	Cloning and engineering of *hgdB* gene for expression of the soluble part
11	5’AGGGTCGGGAGGCGGGTCAGGCGGAGGTAGTGGTGGCGGATCAGGTGGCGGATCCGCACCAGTCAAGACC3’	Cloning and engineering of *hgdB* gene for expression of the soluble part
12	5’TTATTTATCAAAGGTGGAGGAGGCTCTGGTGGAGGCGGTAGCGGAGGCGGAGGGTCGGGAGGCGGGTCAG3’	Cloning and engineering of *hgdB* gene for expression of the soluble part
13	5’AGCCATATGGCAGTAAATAAAGAACGCCAATTATTTATCAAAGGTGGAGGAGGCTCTG3’	Cloning and engineering of *hgdB* gene for expression of the soluble part.
14	5’CAGGCCTTGGCGCGCCTGCAGGCGAGCTCCGTCGACAAGCTTGCGGCCGCAATACTCAAGCACCTGCGGGTGCT GTAGAAGTG3’	Cloning and engineering of *hgdD* gene fragments for expression of the soluble part
15	5’TAATCAATTAGTGGTGGTGGTGGTGGTGGTGGTGCTCGACCTGACTACTAATTAATGCTCTAGAAGTGACAGCAC GTTGTAG3’	Cloning and engineering of *hgdD* gene fragments for expression of the soluble part
16	5’AGTGGTTCCGGAAGTGGTTCCGGAGGGAGAAGGAAAGCTGCTATTC3’	Cloning and engineering of *hgdD* gene fragments for expression of the soluble part
17	5’TCCGGAACCACTTCCGGAACCACTTAATAAAGCGGCCTGTGC3’	Cloning and engineering of *hgdD* gene fragments for expression of the soluble part
18	5’TCCGGTAGTGGATCCGGTAGTGGAGGAGCCGCTAGAGCCAG3’	Cloning and engineering of *hgdD* gene fragments for expression of the soluble part
19	5’TCCACTACCGGATCCACTACCGGACCCCAAGGTTGCTAATGC3’	Cloning and engineering of *hgdD* gene fragments for expression of the soluble part
20	5’CTGTAGCCATGCTAAAGG3’	*hgdB* fw primer
21	5’ATAGCCATCTGGGCTATC3’	*hgdB* rv primer
22	5’TTTGGCAGCATCCCTCAC3’	*hgdB* RT-PCR fw primer
23	5’AAGCGGTAACAGCAACTC3’	*hgdB* RT-PCR rv primer
24	5’GTCAAGGGTGACGGAAGAAC3’	*devB* RT-PCR fw primer
25	5’GGCCTAGCCTTTTTAATTGC3’	*devB* RT-PCR rv primer
26	5’GTTAGCTTAACTGATTTGAG3’	*rnpB* RT-PCR fw primer
27	5’CCCTAGTCCCCAGTCCCCAATCTTG3’	*rnpB* RT-PCR rv primer
28	5’GCATGGCTACAGCTAGTC3’	*hgdC* RT-PCR fw primer
29	5’GGGCTAACTGATTCTACC3’	*hgdC* RT-PCR fw primer
